# Penicilazaphilone C, a New Azaphilone, Induces Apoptosis in Gastric Cancer by Blocking the Notch Signaling Pathway

**DOI:** 10.3389/fonc.2020.00116

**Published:** 2020-02-11

**Authors:** Ming Wang, Huange Zhao, Juanjuan Hu, Zhen Xu, Yingying Lin, Songlin Zhou

**Affiliations:** ^1^Key Laboratory of Tropical Translational Medicine of the Ministry of Education & Hainan Provincial Key Laboratory of Tropical Medicine, Hainan Medical University, Haikou, China; ^2^Health and Family Planning Commission of Wanzai County of Jiangxi Province, Yichun, China; ^3^Department of Medical Insurance Service, Third Affiliated Hospital of Nanchang University, Nanchang, China

**Keywords:** penicilazaphilone C, notch signaling pathway, gastric cancer, apoptosis, molecular mechanism

## Abstract

Penicilazaphilone C (PAC) is a novel azaphilonidal derivative isolated by our group that demonstrates good anticancer activities. Considering that its molecular mechanisms remain largely unknown, here we explore the molecular mechanisms of the anticancer activities of PAC against gastric cancer. The *in vitro* effects of PAC on cell growth, proliferation, and apoptosis were evaluated by MTT, BrdU, MTS, colony formation assays, Hoechst 33258 staining, and flow cytometry. Related proteins were examined by western blotting. Notch receptor expression was analyzed by RT-PCR. *In vivo* antitumor activities of PAC were observed in a nude mouse model. We found that compared to the controls, PAC treatment suppressed cell proliferation and promoted apoptosis in MGC-803 and SGC-7901 cells, and the Notch/PTEN/AKT axis was involved in the activating PAC-induced apoptosis. PAC treatment led to decreased levels of Notch (NTM), NICD, pPTEN, and pAKT compared to controls. PAC-induced inhibition of Notch-related protein expression levels and the resulting apoptosis were reversed by overexpression of Notch1 (NTM) or/and Notch2 (NTM). Moreover, PAC treatment clearly inhibited tumor growth in mice both bearing tumors derived from both MGC-803 and SGC-7901 cells. This work reveals that PAC induces the apoptosis by suppressing activation of Notch receptor proteolytic cleavage and subsequently blocking the PTEN/AKT signaling axis in gastric cancer cells. Thus, PAC is a potential alternative agent for the treatment of gastric cancer.

## Introduction

Gastric cancer (GC) is a very common malignant tumor that is ranked the fourth leading cause of cancer death globally (1), and the morbidity and mortality of GC rank second in China, according to the National Cancer Institute of China (2, 3). Although new anticancer drugs and therapeutic strategies are being increasingly used for GC therapy, overall survival rates of GC are still unsatisfactory, with high incidences and deaths among GC patients, which have not decreased as anticipated (4, 5). Therefore, exploring more effective therapeutic strategies or new anticancer drugs is urgently required to treat this cancer or improve the quality of life of GC patients.

**Figure 1:**
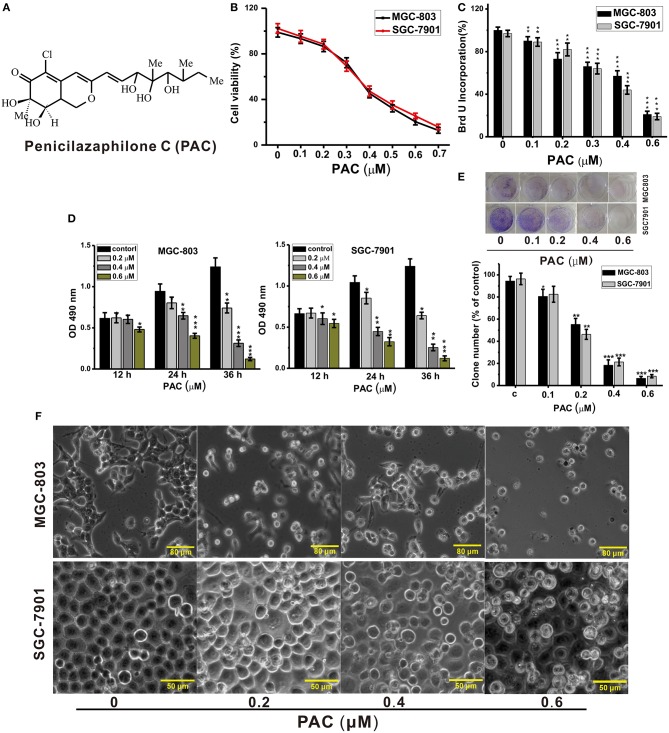
PAC inhibits the proliferation of gastric cancer cells. **(A)** Structure of penicilazaphilone C (PAC). **(B)** MGC-803 and SGC-7901 cells were treated with the indicated concentrations of PAC for 24 h, and cell viability was measured with an MTT assay. **(C)** Cells were treated for 24 h as in **(B)**, and disrupted cell membranes were detected with a BrdU assay. **(D)** Cells were treated as in **(B)** for 12, 24, and 36 h, and MTS-incorporating live cells were detected with an MTS proliferation assay. **(E)** Cells were treated as in **(B)** for 14 days, and live cells were detected with a colony formation assay. **(F)** Cells were treated as in **(B)** for 24 h; cell morphology was observed under an inverted phase-contrast microscope and images were obtained. Data are expressed as the mean ± SD; *P < 0.05, **P < 0.01, ***P < 0.001.

Penicilazaphilone C (PAC, Figure 1A) is a novel natural product that was isolated by our research group from the marine fungus *Penicillium sclerotiorum* M-22 (6). PAC is a novel azaphilone with a special pyranoquinone bicyclic core structure (6). Previous studies have shown that azaphilone molecules exert a variety of important biological activities (7–9). Indeed, PAC showed highly selective cytotoxicity in GC cells in our previous study (6). However, the molecular mechanism by which PAC inhibits GC cells remains unclear.

The Notch signaling pathway is widely distributed among vertebrates and invertebrates and is highly evolutionarily conserved (10). Notch signaling affects multiple normal morphologenic processes, including the differentiation of pluripotent progenitor cells, apoptosis, cell proliferation, and cell boundary formation (11, 12). Humans and rodents have four Notch receptors (Notch l−4) and five ligands (Jagged 1, 2, Delta-like 1, 3, 4,) (13). The Notch receptor is composed of an extracellular region, a single transmembrane region, and an intracellular region containing an ankyrin domain and a RBP-JK-associated molecule domain (14). Notch signaling is activated by interaction of a ligand from adjacent cells with the Notch receptor (14). Two cleavage events release the intracellular domain of the Notch receptor (NICD) into the cytoplasm, after which it enters the nucleus and binds to the transcription factor CSL to activate the NICD/CSL transcription complex, thereby inducing expression of target genes, such as HES, HEY, and HERP, and exerting biological effects (13). In this study, we investigated the therapeutic effect of PAC on GC in detail to identify the underlying mechanism of PAC-mediated inhibition of GC cell proliferation.

## Materials and Methods

### Cell Lines and Culture

The human GC cell lines MGC-803, HGC-27, AGS, MKN-45, and SGC-7901 and the normal gastric mucosa cell line GES-1 were obtained from ATCC (Manassas, USA). The cells were cultured in DMEM or RPMI-1640 (HyClone, USA), supplemented with 10% fetal bovine serum, 100 μg/mL streptomycin, and 100 U/mL penicillin (HyClone, USA) at 37°C in a humidified incubator containing 5% CO_2_. Cells were passaged at 80–90% confluence and were used for experiments in the exponential growth phase.

### Cell Viability and Proliferation Assays

Cells were incubated in 96-well plates and exposed to the indicated concentration of PAC (0.1, 0.2, 0.3, 0.4, 0.5, 0.6, 0.7 μM) or vehicle control (DMSO) for 24 h. Cell viabilities were evaluated with an MTT assay (Sigma, USA). In brief, 10 μL of 5 mg/mL MTT was added to each well and incubated for 4 h. The medium was replaced with 150 μL of DMSO to dissolve the crystal formazan dye and absorbance was detected at 540 nm using an ELX808IU Microplate Reader (BioTek, USA).

Cells were seeded in 96-well plates and exposed to the indicated concentration of PAC (0.1, 0.2, 0.3, 0.4, 0.6 μM) or vehicle control (DMSO) for 24 h. Cell proliferation was measured with a BrdU assay (Abcam UK): BrdU (10 μM) was added to each well and incubated for 12 h, and the BrdU signal was calculated after absorbance detection at 450 nm.

Cells were plated in 96-well plates and exposed to the indicated concentrations of PAC (0.2, 0.4, 0.6 μM) or vehicle control (DMSO) for 12, 24, and 36 h. Cell viability was examined with an MTS assay (Sigma, USA), 20 μL of MTS was added to each well and incubated for 1 h, after which the absorbance of the MTS signal was calculated after absorbance detection at 490 nm.

### Colony Formation Assay

Cells were treated with the indicated amount of PAC (0.1, 0.2, 0.4, 0.6 μM) or vehicle control (DMSO) for 24 h and cultured at a density of 500 cells/well in 6-well plates. The medium was changed every 3 d. Two weeks later, cell colonies were stained with Giemsa Stain Solution (Solarbio, CN). Visible colonies were photographed and counted using a GelDocTMXR^+^ Molecular Imager system (BioRad, USA).

### Hoechst 33258 Staining

The morphology of apoptotic cells was observed using a Hoechst 33258 Staining Kit following the manufacturer's instructions and an IX73-AF12/PH fluorescence microscope (Olympus, JAN).

### Flow Cytometry

Cells were incubated in 6-well plates and exposed to the indicated concentrations of PAC or vehicle control for 24 h. Apoptosis and cell cycle analyses were performed using an Annexin-V FITC/PI Staining Kit and Cell-Cycle Detection Kit (KeyGEN-Biotech, CN) following the manufacturer's instructions. All data were evaluated by FlowJo software (BD Biosciences, USA).

### Real-Time RT-PCR Analysis

Total RNA was extracted from cells with a Trizol Reagent Kit (Invitrogen) at the indicated time points, and Notch1, Notch2, and control β-actin complementary DNA syntheses were performed with a RevertAid First Strand cDNA Synthesis Kit (Fermentas, USA). RT-PCR was carried out as reported previously (15). The primer pairs used are listed in Supplementary Table 1.

### Western Blotting

Samples were prepared for western blotting according to the protocol by Sun et al. (16). Primary antibodies against β-actin, Notch1, Notch2, N1ICD, N2ICD, HES1, PTEN, p-PTEN, AKT, p-AKT, Bax, Bcl-2, Caspase-3, and Caspase-9 and the secondary antibody (goat anti-rabbit antibody conjugated to horseradish peroxidase) were all purchased from Cell Signaling (Massachusetts, USA).

### Plasmids and Cell Transfection

Notch1-NTM (NM017617) and Notch2-NTM (VH879689) expression plasmids were constructed by Vigene Biosciences (Jinan, CN). The plasmids were transfected into cells using Lipofectamine 2000 following the manufacturer's recommended protocols.

### Observation of the Antitumor Effect *in vivo*

Animal experiments were approved by the Animal Care and Use Committee of Hainan Medical College and abided by animal protocols (grant number: HYLL-2019-033). MGC-803 and SGC-7901 tumor models were generated using male BALB/c-nude mice at 7 weeks of age. The mice were subcutaneously inoculated in the right inguinal region with the corresponding GC cells (1 × 10^6^). When the tumor was visible (~50 mm^3^ at ~7 d), the mice were randomly divided into two groups (5 in each group) for treatment with 0.1 mL DMSO (PAC 0 mg/kg) or PAC (30 mg/kg) via the tail vein once every 3 d. The treatments were performed 7 times, and the mice were observed for 4 weeks. Tumor volumes were assessed every week using the following formula: tumor volume = (width^2^ × length)/2.

### Statistical Analysis

Statistical analysis was carried out with Prism 7. Differences were analyzed using one-way ANOVA or a two-sample equal-variance Student's *t-*test. Data are expressed as the mean ± SD, and *P* < 0.05 was deemed to be statistically significant.

## Results

### Penicilazaphilone C (PAC) Suppresses the Proliferation of GC Cells

To explore the anticancer activities of PAC, we examined cell growth and proliferation in two GC cell lines, MGC-803 and SGC-7901. The cells were exposed to different amounts of PAC for the indicated durations, followed by analysis by MTT, BrdU, MTS, and colony formation assays to examine whether PAC affected cell viability and proliferation. MTT assays showed that viability in both MGC-803 and SGC-7901 was dose-dependently suppressed by PAC compared to that in controls (Figure 1B). Subsequently, similar inhibitory effects of PAC treatment for 24 h were observed based on BrdU assays (Figure 1C). Moreover, the results of MTS assays indicated that the cytotoxic effects of PAC on these two GC cells line not only increased with increasing dose but also with exposure time (Figure 1D). Additionally, microscopy images showed obvious cellular shrinkage after PAC treatment, with significantly decreased cellular attachment in comparison with controls (Figure 1F). Furthermore, colony formation assays suggested that PAC treatment markedly suppressed proliferation in MGC-803 and SGC-7901 cells compared to controls (Figure 1E). We also assessed whether PAC is toxic to normal human gastric mucosal cells (GES-1), and the results showed that PAC at <1.6 μM was not cytotoxic to normal gastric cells; the dose that produced obvious cytotoxicity was ~3.2 μM, more than eightfold the dose used in our assays (Supplementary Figure 1). These findings indicate that PAC specifically suppresses the viability and growth of GC cells. The IC_50_ of PAC was ~0.4 μM in both MGC-803 and SGC-7901, as shown in Figure 1B.

### PAC Induces Apoptosis in GC Cells

**Figure 2:**
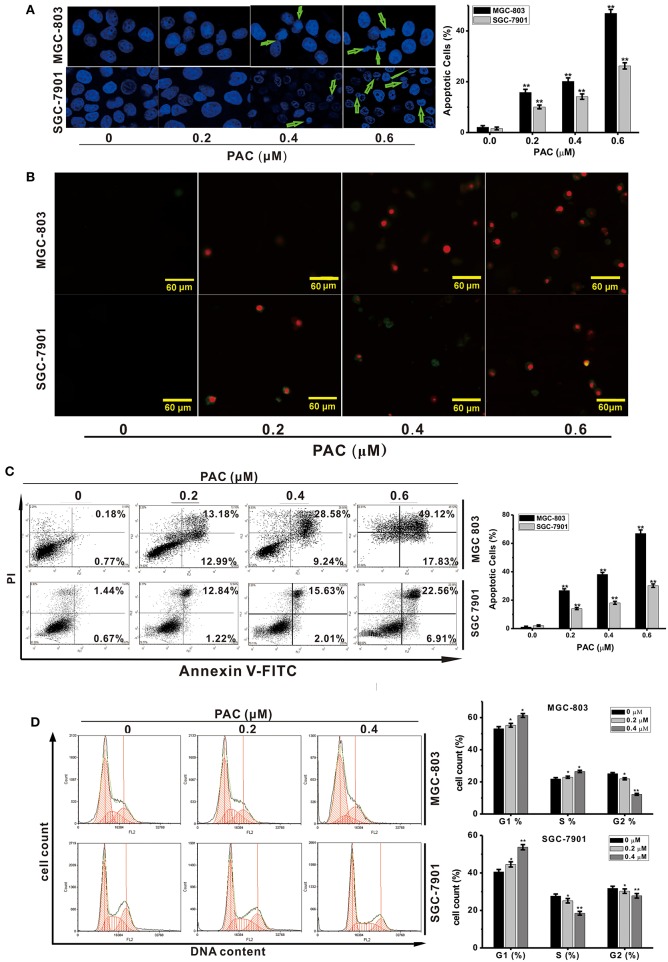
PAC induces apoptosis in gastric cancer cells. **(A)** MGC-803 and SGC-7901 gastric cancer cells were treated with the indicated concentrations of PAC for 24 h and stained with Hoechst 33258. Typical apoptotic morphology was observed in treated cells compared to untreated cells. **(B)** Cells were treated as in **(A)** for 24 h and stained with annexin V/PI. **(C)** Cells were treated as in **(A)** for 24 h, apoptotic cells were stained by PI/annexin-V, and flow cytometry was performed. **(D)** Cells were treated as in **(A)** for 24 h, stained with PI and subjected to flow cytometry to analyze the distribution in each phase of the cell cycle. Data are expressed as the mean ± SD; *P < 0.05, **P < 0.01.

Next, we explored whether inhibition of cell proliferation after PAC treatment was accompanied by apoptosis. We evaluated changes in nuclear morphology and the percentage of apoptotic MGC-803 and SGC-7901 cells by using a Hoechst 33258 staining solution. After PAC treatment, chromatin condensation and cell nuclear shrinkage, which are typical apoptotic morphological characteristics, were significantly increased in the two GC cell lines compared to the controls (Figure 2A). Similar, apoptotic characteristics in GC cells were observed based on annexin V/PI staining after PAC treatment (Figure 2B). To further confirm the apoptotic effects of PAC on MGC-803 and SGC-7901 cells, we performed flow cytometry after annexin V-FITC/PI staining and found that PAC caused both early and late apoptosis in a dose-dependent manner. As shown in Figure 2C, the percentage of apoptotic MGC-803 cells was 1.07% in the control group and reached 26.78 ± 1.21, 38.15 ± 1.36, and 66.92 ± 2.53% in the treatment groups; for SGC-7901 cells, the percentage of apoptotic cells was 2.08% in the control group and 14.07 ± 0.75, 18.13 ± 1.07, and 30.23 ± 1.23% in the treatment groups. These results prove that the percentage of apoptotic cells increased markedly with increasing doses of PAC. Our findings indicate that PAC can induce apoptosis in GC cells.

### PAC Causes Cell-Cycle Arrest in G1 Phase

To investigate whether the inhibition of cell proliferation due to PAC treatment was associated with cell cycle arrest, the influence of PAC on the cell cycle in MGC-803 and SGC-7901 cells was examined. Upon PAC treatment, G1-phase cells were obviously increased compared with those in the control group. As shown in Figure 2D, the percentage of MGC-803 cells in G1 and S phases was increased compared to those in the control group; however, only the percentage of SGC-7901 cells in G1 phase was increased. Our findings show that PAC arrested GC cells in the G1 phase of the cell cycle.

### Expression of Notch1 and Notch2 in GC Cell Lines

To evaluate Notch signaling activation in GC cells, we examined relative expression of Notch1 and Notch2 at the mRNA and protein levels in five GC cell lines and GES-1 cells (Supplementary Figures 2a,b). Expression of both Notch1 and Notch2 mRNA and protein was obvious in the five GC cell lines. Compared to GES-1 cells, Notch1 RNA and protein expression was higher in the various gastric cancer cells, whereas Notch2 expression was variable. For example, Notch2 expression was lower in MKN-28, MKN-45 and MGC-803 cells but higher in SGC-7901 and AGS compared to GES-1 cells, which was consistent with previous studies (17, 18), Therefore, we selected a cell lines with high Notch2 expression (SGC-7901) and a cell lines with low Notch2 expression (MGC-803) as the for subsequent experiments.

### PAC Induces Dephosphorylation of AKT and Blocks Notch Signaling in GC Cells

**Figure 3:**
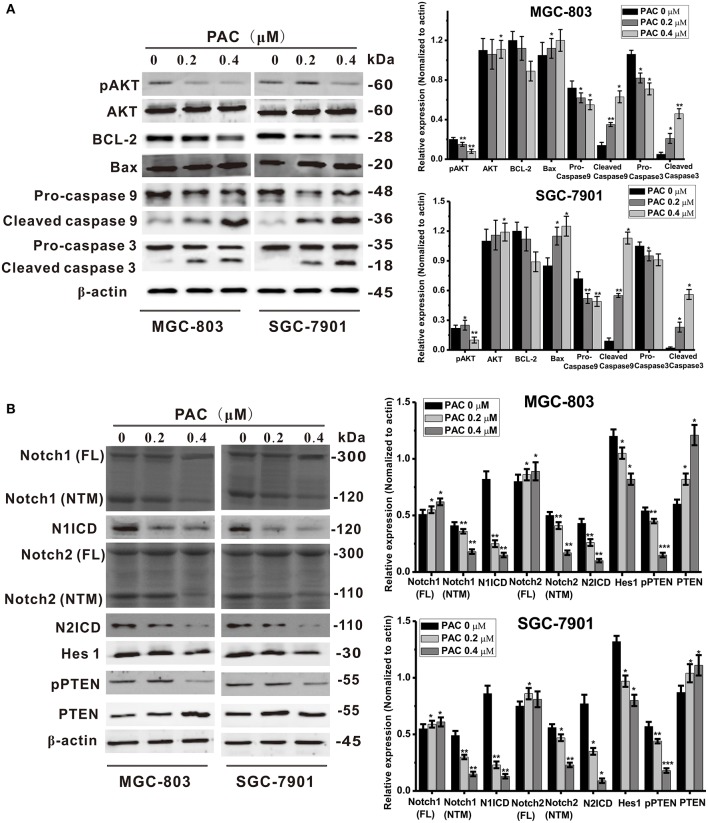
PAC induces apoptosis through inhibition of the Notch/PTEN/AKT pathway. **(A)** Expression levels of apoptosis-related proteins were detected by western blot analysis after MGC-803 and SGC-7901 gastric cancer cells were treated with the indicated concentrations of PAC. **(B)** Expression levels of Notch signaling pathway-related proteins were detected by western blot analysis after MGC-803 and SGC-7901 gastric cancer cells were treated with the indicated concentrations of PAC. Data are expressed as the mean ± SD; *P < 0.05, **P < 0.01, ***P < 0.001.

Recent reports have revealed that the PTEN/AKT signaling pathway is involved in the regulation of apoptosis, and there are many reports that antitumor drugs induce apoptosis by inhibiting this pathway (19, 20). AKT is an effector molecule downstream of PTEN (21). Simultaneous phosphorylation of Thr308 and Ser473 is required for complete activation of AKT, and activated AKT can inhibits pro-apoptotic factors, such as Bax, Bad, and caspase-9, and activate anti-apoptotic factors, such as Bcl-2 and Bcl-xL (15). To examine whether PAC-induced apoptosis is stimulated by a PTEN/AKT-related pathway in GC cells, PTEN/AKT axis-related proteins were detected by western blotting. As shown in Figure 3A, AKT phosphorylation levels were significantly decreased upon PAC treatment in a dose-dependent manner compared to controls, though total AKT expression was increased in a dose-dependent manner. In addition, apoptosis was observed after PAC treatment, which was confirmed by increased cleavage of caspase-9 and−3 as well as the increased ratio of Bax/Bcl-2 at 24 h compared to controls.

Because Notch signaling impacts the PTEN/AKT signaling axis, we also assessed the expression of PTEN- and Notch-related proteins. As shown in Figure 3B, compared to controls, the protein content of full-length Notch1 and Notch2 increased slightly in both GC cell lines, but the transmembrane fragments of Notch1 (Notch1-NTM) and Notch2 (Notch2-NTM) were notably decreased and dramatically decreased in N1ICD and N2ICD after 24 h of PAC treatment. Consistently, the expression levels of the target gene Hes1 were also significantly decreased in the PAC-treated group compared to those in the controls. In contrast, as a negative regulator downstream of PTEN, PAC slightly increased PTEN protein expression, though PTEN phosphorylation was significantly decreased compared to the controls. We also examined changes in the levels of the Notch ligands Jagged 1, Jagged 2, and DLL4 and found that the protein expression of these ligands did not change significantly upon PAC treatment (Supplementary Figure 3). These findings suggest that PAC treatment may block the initial proteolytic events in Notch receptor activation.

### Overexpression of the Transmembrane Fragment of Notch Reverses the Effect of PAC on Apoptosis

**Figure 4:**
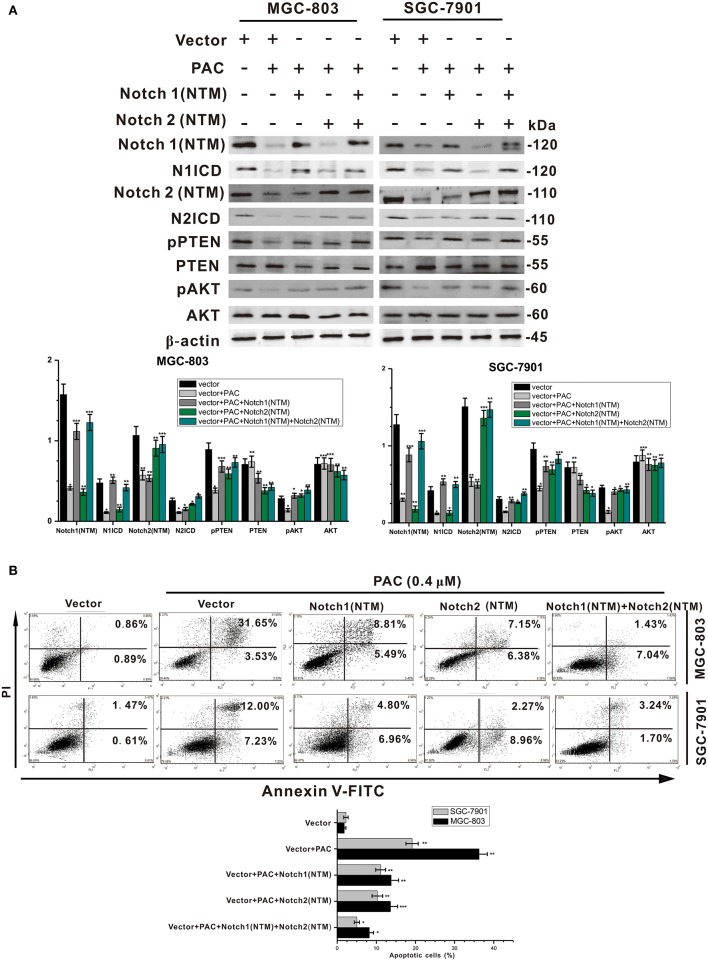
PAC induces apoptosis in gastric cancer cells through blockage of the PTEN/AKT axis. **(A)** Cells were treated with or without PAC (0.4 μM) in combination with transfection of the Notch1 (NTM) plasmid and/or Notch2 (NTM) plasmid for 24 h. Notch1 (NTM), N1ICD, Notch2 (NTM), N2ICD, Hes1, PTEN, phosphorylated PTEN (pPTEN), AKT, and phosphorylated AKT (pAKT) were detected by western blotting. **(B)** Cells were treated with or without PAC (0.4 μM) in combination with transfection of the Notch1 (NTM) plasmid and/or Notch2 (NTM) plasmid for 24 h, apoptotic cells were stained by PI/annexin-V, and flow cytometry was performed. Data are expressed as the mean ± SD; *P < 0.05, **P < 0.01, ***P < 0.001.

To examine the mechanism underlying the suppression of GC cell proliferation and the induction of apoptosis by PAC, GC cells were transfected with a Notch1 (NTM) and/or Notch2 (NTM) overexpression plasmid, with contrasting expression profiles for proteins in the Notch/PTEN/AKT axis. As presented in Figure 4A, overexpression of Notch1 (NTM) and/or Notch2 (NTM) resulted in a significant increase in NICD, Hes1, pPTEN, and pATK in both MGC-803 and SGC-7901 cells compared to controls. Conversely, protein expression of PTEN and AKT was slightly decreased in the overexpression groups compared to that in the controls. Moreover, compared to controls overexpression of Notch1 and/or Notch2 notably reduced PAC-induced apoptosis in the two GC cell lines transfected with plasmids (Figure 4B). Our results strongly indicate that PAC induces apoptosis in GC cells by blocking the initial proteolytic cleavage of the Notch receptor and release of NICD into the cytoplasm.

### PAC for Tumor Treatment *in vivo*

**Figure 5:**
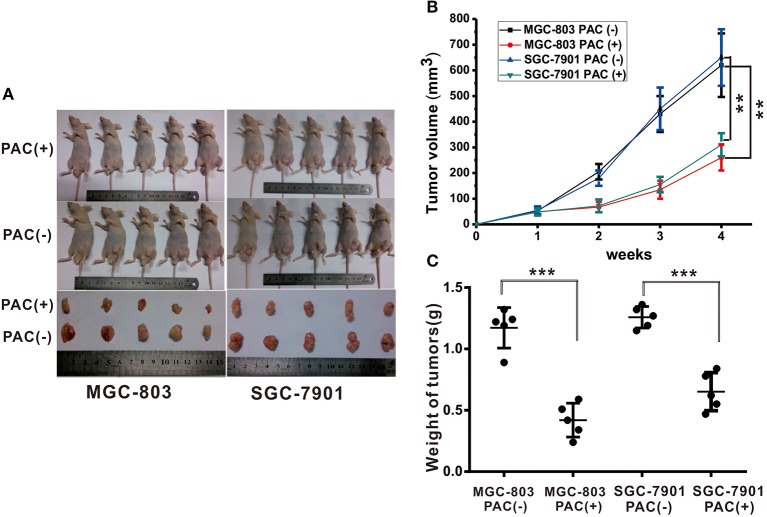
PAC induced suppression of gastric tumor growth. Human MGC-803 and SGC-7901 gastric cancer cells were injected into the right flanks of Nu/Nu mice. When tumor volumes reached 50 mm^3^, the mice were treated with the indicated formulations every 3 days for 7 total treatments. **(A)** Pictures of mice taken after 4 weeks of treatment with the indicated therapies. **(B)** Tumor growth curves of all right and left flank tumors with the indicated treatments. **(C)** Tumor weight curves for the indicated therapies. Data are expressed as the mean ± SD; **P < 0.001, ***P < 0.001.

To examine the antitumor activities of PAC *in vivo*, MGC-803 and SGC-7901 cells were inoculated into BALB/c-nude mice. This experiment clearly demonstrated that PAC is an effective *in vivo* antitumor drug. As depicted in Figures 5A,B, PAC treatment significantly suppressed tumor growth compared to that in the control group. Figure 5C also shows that the tumors in the PAC-treated group, which were collected at 28 d, were smaller and weighed less than did those in the control group.

## Discussion

Despite the significant contributions of tumor chemotherapy and resection to gastric carcinoma treatment, genetic variation, and drug resistance reduce the efficacy of these treatments (22). The natural products of marine fungi are rich in unique structural types (23), and PAC is a novel natural compound with a special azaphilone chemical structure isolated from the marine fungus *P. sclerotiorum* M-22 by our research group that exhibits anticancer activity in several cancer cell lines. Although PAC exhibits a marked anticancer effect, it is a new azaphilone structure, and the ways by which PAC regulates cancer programmes remains unknown. In this study, we attempted to elucidate the underlying mechanisms by which PAC affects GC cells.

As the most notable hallmark of tumor cells is uncontrolled growth (24), we first examined whether PAC is able to inhibit cancer cell growth (proliferation) by performing MTT, BrdU, MTS, and colony formation assays. The results showed that PAC obviously inhibited cell proliferation in MGC-803 and SGC-7901 cells in a dose- and time-dependent manner. Next, we found that PAC induces apoptosis and cell cycle arrest in the G1 phase by restraining AKT signaling, which is involved in cell proliferation and apoptosis. As expected, pAKT levels were decreased upon PAC treatment compared to that in controls. In contrast, the pro-apoptotic factors cleaved caspase−9 and−3 and the Bax/Bcl-2 ratio was increased in a dose-dependent manner after PAC treatment compared to controls. Notch signaling is predicted to be a target of PAC based on our endogenous tumor network model, and the regulation of Notch signaling plays a key role in tumor progression. Previous reports have shown that Notch signaling inhibits transcription of PTEN and activates the PI3K/AKT pathway (21, 25). Kim et al. (15) reported that the Notch-targeted protein Hes-1 suppresses PTEN expression through induced reversible phosphorylation of PTEN C-terminal sites and activates PI3K/AKT signaling in GC cells. Subsequently, we assessed the influence of PAC on Notch-related signaling. Upon PAC treatment, phosphorylation of PTEN and expression of Notch1 (NTM), Notch2 (NTM), N1ICD, N2ICD, and Hes1 were markedly decreased but the content of PTEN increased compared to controls. Additionally, we found that PAC treatments slightly enhanced the expression of the three ligands, full-length Notch1 and Notch2 compared to controls. These findings suggest that PAC blocks the first proteolytic events in Notch receptor activation. Furthermore, compared to controls, PAC-induced inhibition of Notch-related protein expression levels was promoted by overexpression of Notch1 (NTM) and/or Notch2 (NTM), though overexpression of Notch1 (NTM) and/or Notch2 (NTM) also suppresses the apoptosis caused by PAC. Our results strongly suggest that PAC treatment may inhibit the enzymatic cleavage that occurs during Notch receptor activation and then block NICD binding to target genes, ultimately exerting biological effects.

The binding of Notch receptors and ligands in adjacent cells triggers the Notch signaling pathway (26). As a result of binding, the receptor undergoes sequential proteolytic cleavages. First, the extracellular fragment is liberated from the extracellular surface by α-secretase, and then the NICD is released into the cytoplasm by γ-secretase (27); these two secretases belong to the ADAM (a disintegrin and metalloproteinase) endopeptidase family (28). The results of this experiment indicate that PAC can inhibit Notch receptor proteolytic activation by α-secretase, subsequently blocking the Notch pathway. However, it remains unclear how PAC interacts with α-secretase to inhibit the activation of α-secretase. It is also not known whether PAC can inhibit other ADAM endopeptidases. Thus, the mechanisms underlying PAC-mediated α-secretase activities are still poorly understood, and further research is needed.

**Figure 6:**
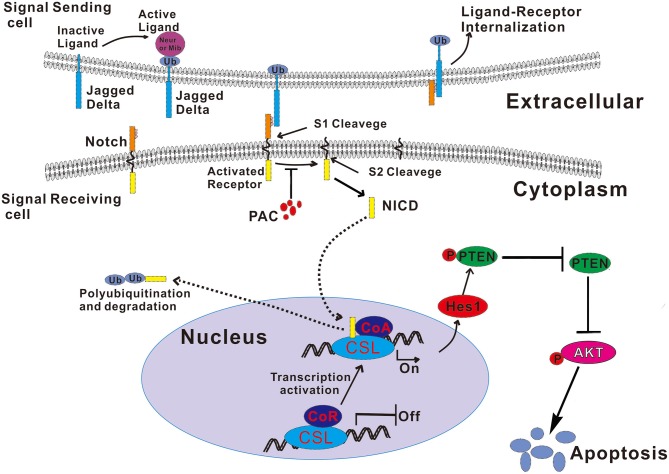
Schematic illustration of the mechanism by which PAC induces apoptosis by blocked the Notch/PTEN/AKT axis in gastric cancer cells. Ub, ubiquitin.

In summary, this study demonstrates that PAC is a potential therapy for GC. PAC induces apoptosis by blocking the proteolytic cleavage of the activated Notch receptor and further influences the PTEN/AKT axis. Our experimental data reveal the molecular mechanism underlying the anticancer activity of PAC (summarized in Figure 6) and strongly suggest that PAC, as an inhibitor of Notch signaling, is a potential alternative drug for the treatment of GC patients.

## Data Availability Statement

All datasets generated for this study are included in the article/Supplementary Material.

## Ethics Statement

Animal experiments and protocol followed the guidelines and regulations set by the Animal Care and Use Committee of Hainan Medical College (Grant number: HYLL-2019-033).

## Author Contributions

MW: data analysis and interpretation. HZ: data collection and analysis. YL and ZX: data collection. JH: manuscript writing and polishing. SZ: conception and design, data analysis and interpretation, and writing. All authors read and approved the final manuscript.

### Conflict of Interest

The authors declare that the research was conducted in the absence of any commercial or financial relationships that could be construed as a potential conflict of interest.
