# Supplementary material for: Penicilazaphilone C, a New Azaphilone, Induces Apoptosis in Gastric Cancer by Blocking the Notch Signaling Pathway
Source: Front Oncol. 2020 Feb 11;10:116. doi: 10.3389/fonc.2020.00116 (PMC7026506; doi:10.3389/fonc.2020.00116)
Supplement: Supplementary file 1 [file Table_1.DOCX]

Supplementary Material

# Supplementary Tables

# Supplementary Tables 1. RT-PCR Primers

| **Gene** | **Primer sequences** |
| --- | --- |
| ***Notch1*** | F : 5’ - GAGGCGTGGCAGACTATGC - 3’  GCAGTTGTGCTCCTGAAGAA |
|  | R : 5’ - CTTGTACTCCGTCAGCGTGA - 3’  CGGGCGGCCAGAAAC |
| ***Notch2*** | F : 5’ - TATTGATGACTGCCCTAACCACA - 3’ |
|  | R : 5’ - ATAGCCTCCATTGCGGTTGG - 3’ |
| β-actin | ***F : 5’ -*** TGGCACCCAGCACAATGAA ***- 3’*** |
|  | ***R : 5’ -*** CTAAGTCATAGTCCGCCTAGAAGCA ***- 3’*** |

## Supplementary Figures

**
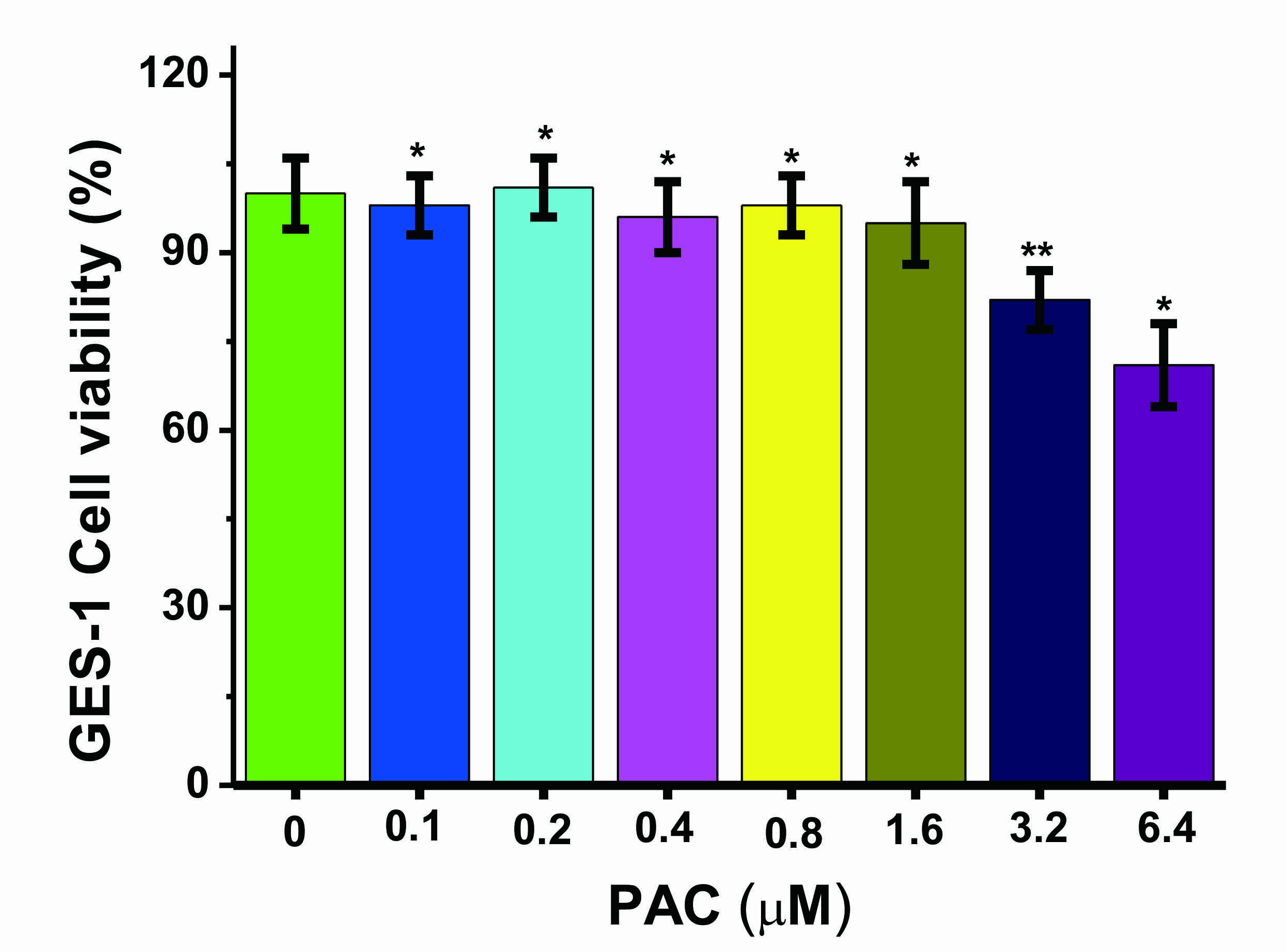
**

**Supplementary Figure 1.** **PAC inhibits proliferation of normal human gastric mucosa cell (GES-1).** GES-1 cells were treated with indicated concentrations of PAC for 24 h. Cell viability was examined by MTT assay. The number from the DMSO (0 μM) group was counted as 100 %, and those of other groups were relative to that. Data are expressed as mean ± SD; * *P* < 0.05, ** *P* < 0.01, *** *P* < 0.001.


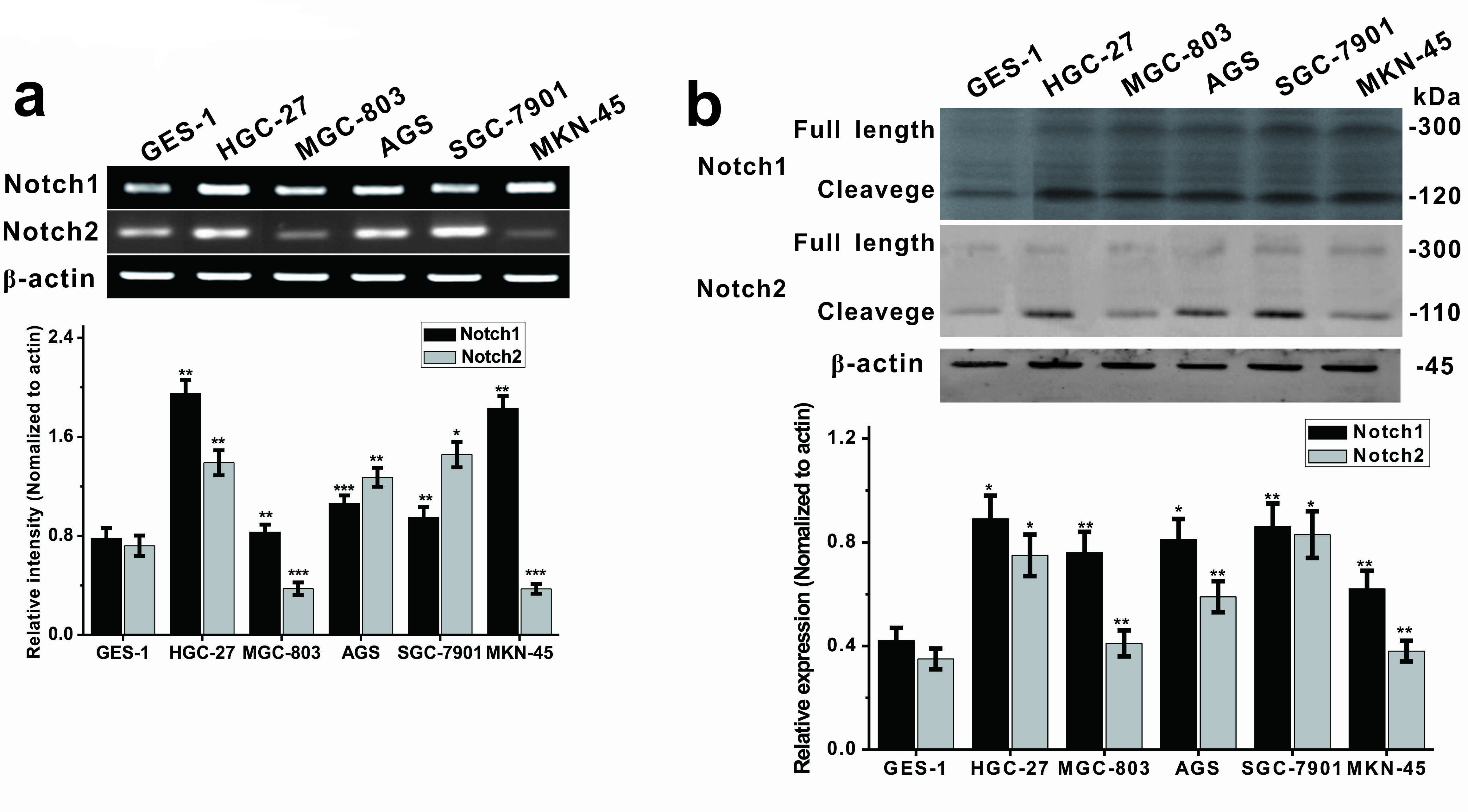


**Supplementary Figure 2. Expression mRNA and protein of Notch 1 and Notch 2 in GC cell lines and GES-1 cells.** (**a**) mRNA Expression levels of Notch1 and Notch2 gene was detected by real-time fluorescence quantitative-PCR (RFQ-PCR); (**b**) Protein expression levels of Notch1 and Notch2 was detected by western blot. Data are expressed as mean ± SD; * P < 0.05, ** P < 0.01.


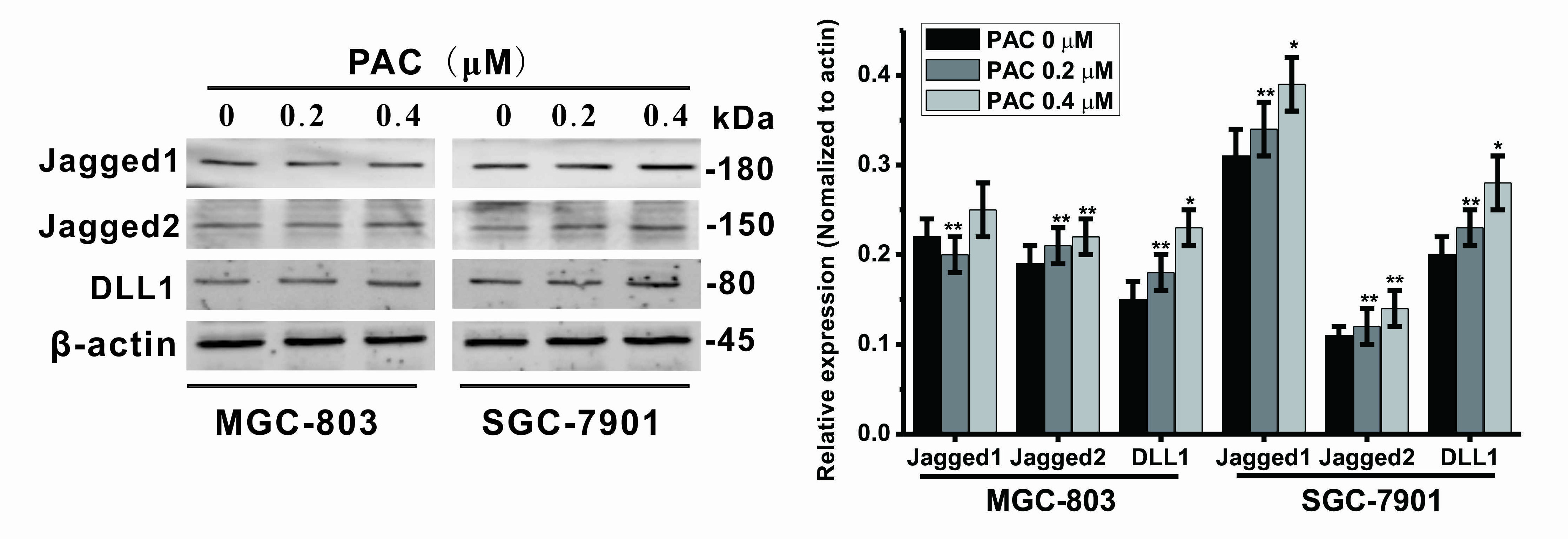


**Supplementary Figure 3. Expression levels of Notch ligands (Jagged1, Jagged2 and DLL1) proteins were detected by western blot analysis after MGC-803 and SGC-7901 gastric cancer cells were treated with indicated concentrations of PAC**. Data are expressed as mean ± SD; * P < 0.05, ** P < 0.01, *** P < 0.001.
